# Surface imaging‐based analysis of intrafraction motion for breast radiotherapy patients

**DOI:** 10.1120/jacmp.v15i6.4957

**Published:** 2014-11-08

**Authors:** David B. Wiant, Stacy Wentworth, Jacqueline M. Maurer, Caroline L. Vanderstraeten, Jonathon A. Terrell, Benjamin J. Sintay

**Affiliations:** ^1^ Department of Radiation Oncology Cone Health Cancer Center Greensboro NC USA

**Keywords:** breast radiotherapy, intrafraction motion, surface imaging

## Abstract

Breast treatments are becoming increasingly complex as the use of modulated and partial breast therapies becomes more prevalent. These methods are predicated on accurate and precise positioning for treatment. However, the ability to quantify intrafraction motion has been limited by the excessive dose that would result from continuous X‐ray imaging throughout treatment. Recently, surface imaging has offered the opportunity to obtain 3D measurements of patient position throughout breast treatments without radiation exposure. Thirty free‐breathing breast patients were monitored with surface imaging for 831 monitoring sessions. Mean translations and rotations were calculated over each minute, each session, and over all sessions combined. The percentage of each session that the root mean squares (RMS) of the linear translations were outside of defined tolerances was determined for each patient. Correlations between mean translations per minute and time, and between standard deviation per minute and time, were evaluated using Pearson's *r* value. The mean RMS translation averaged over all patients was 2.39mm±1.88mm. The patients spent an average of 34%, 17%, 9%, and 5% of the monitoring time outside of 2 mm, 3 mm, 4 mm, and 5 mm RMS tolerances, respectively. The RMS values averaged over all patients were 2.71mm±1.83mm, 2.76±2.27, and 2.98mm±2.30mm over the 5th, 10th, and 15th minutes of monitoring, respectively. The RMS values (r=0.73,p=0) and standard deviations (r=0.88,p=0) over all patients showed strong significant correlations with time. We see that the majority of patients' treatment time is spent within 5 mm of the isocenter and that patient position drifts with increasing treatment time. Treatment length should be considered in the planning process. An 8 mm margin on a target volume would account for 2 SDs of motion for a treatment up to 15 minutes in length.

PACS numbers: 87.53.Jw, 87.53.Kn, 87.56.Da, 87.63.L‐

## I. INTRODUCTION

As with many disease sites, breast radiation is becoming increasingly complex as we strive for an improved therapeutic ratio. Hypofractionated courses, field in fields, electronic compensated fields, intensity‐modulated radiation therapy (IMRT), and volumetric‐modulated arc therapy (VMAT) are all becoming more prevalent as treatment delivery and image guidance systems continue to develop and become available in the clinic. All of these techniques are predicated on accurate patient position and reliable immobilization. A great deal of work has been done in the area of interfraction setup variability. It has been definitively demonstrated that electronic portal images are capable of reducing interfraction setup variations for breast radiotherapy to <5mm.^1^, ^2^, ^3^, ^4^, ^5^, ^6^, ^7^, ^8^ Intrafraction motion during breast radiotherapy has also been widely investigated using electronic portal images.^1^, ^2^, ^3^, ^7^, ^8^, ^9^ However, intrafraction motion studies with electronic portal images are fundamentally limited by exposure of a patient population expected to have long‐term survival to unnecessary ionizing radiation. Both the number of patients and the number of portal images are limited by these practical radiation safety concerns. The number of patients in these studies cited above ranged from 8 to 20. The highest intrafraction motion sampling rates came from studies that recorded treatment field images in cine mode during actual treatment delivery.^2^, ^3^, ^9^ These groups reported sampling rates on the order of two images/s, with 4–25 images recorded per treatment field. Therefore, these monitoring sessions were limited to < 15 s of continuous sampling at a single planar view. Cine treatment recordings provide valuable information, but with limited sampling time and a single 2D viewing angle, they are not able to fully characterize 3D motion for a complex treatment session that might last 15 minutes or more.

Recently, 3D surface imaging has become available as a tool to aid in setup and to evaluate motion.^10^, ^11^, ^12^ Three‐dimensional surface imaging uses nonionizing radiation, so there are no safety‐related limits on sampling rate and frequency. Surface imaging is capable of providing full 3D monitoring of patient position in near real time. The commercially available AlignRT (VisionRT, London, UK) 3D surface imaging system has shown the abilities to detect submillimeter changes in patient position^10^, ^12^, ^13^, ^14^, ^15^, ^16^, ^17^ and to accurately localize breast patients for free breathing^18^, ^19^, ^20^, ^21^ and deep inspiration breath‐hold treatments^22^, ^23^ to better than 5 mm. These works have shown that AlignRT is capable of accurately and reproducibly positioning breast tissue relative to treatment isocenter for initial patient positioning and, prior to beam‐on, for breath‐hold treatments. Based on this body of work, it is reasonable to conclude that AlignRT can provide accurate intrafraction motion information over entire courses of treatment for large numbers of patients at high sampling rates. In this work, we examine the AlignRT real‐time patient position measurements for the entire course of treatment for 30 breast patients treated at our institution. These patients were monitored over 831 fractions for a total of 4,747 min, with an average monitoring time of 5.7 min. These real time 3D measurements were evaluated in relation to time on the treatment table and fraction number to evaluate patient movement over the course of treatment. To the best of our knowledge these efforts represent the largest and most thorough evaluation of intrafraction breast motion and, as such, they may provide valuable insights into modern breast planning, setup, and treatment delivery.

## II. MATERIALS AND METHODS

### A. Surface imaging system validation

The stability and accuracy of the surface imaging system were tested with a phantom created to mimic the shape of a torso and to be visible on the surface imaging system. A CT scan was acquired on a Philips Big Bore (Philips Healthcare, Andover, MA) scanner with nominal 3 mm slice thickness and the field of view set to include the patient and immobilization device. A contour of the external surface of the phantom was created in MIM Maestro (MIM Software, Cleveland, OH) and sent in DICOM format to the AlignRT system.

The AlignRT system allows a reference surface to be created from either the imported DICOM‐format body surface structure created in the treatment planning process (DCMREF) or from a static AlignRT image captured with the patient in the treatment position (VRTREF).^(12)^ A DCMREF surface was created for the phantom from the imported structure. Once the phantom was aligned to the DCMREF surface, a VRTREF surface was also acquired.

Each surface was monitored for 15 min intervals. The mean position of the phantom and the range of readouts over the time period were analyzed as a check of stability. The phantom was then moved to 30 unique positions between 0–5 mm in the vertical, longitudinal, and lateral dimensions to evaluate the ability of the surface imaging system to track positional changes for each type of reference surface. The positioning was performed via treatment couch movements. The accuracy of the couch readout was previously determined to be ±0.1mm. The difference between the expected position and the position measured by the surface imaging system were recorded.

### B. Patient measurements

Thirty patients who received breast radiotherapy were retrospectively reviewed for this study. All of the patients had intact breasts. Twenty‐nine of the patients were treated with tangent and reduced fields while free breathing. One of the patients was treated free breathing with multiple IMRT fields. All of the patients were immobilized on a commercial breast board (QFix, Avondale, PA) at a 10°–15° angle with both arms raised above their heads. All of the patients underwent computed tomography scans on a Philips Big Bore with nominal 3 mm slice thickness and the field of view set to include the patient and immobilization device. The patients were all contoured in MIM Maestro and the plans were created in the Eclipse treatment planning system (Varian, Inc., Palo Alto, CA). The plans and structures were sent in DICOM format to the AlignRT system for treatment preparation. All treatments were delivered on a Varian TrueBeam or TrueBeam STx. All patients received port images at the initial pretreatment verification day and then once per week.

**Figure 1:**
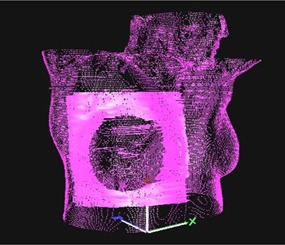
The striated pink surface represents a reference surface generated using the body structure from the treatment plan. The solid pink surface is the ROI used for AlignRT registration. The ROI includes the ipsilateral chest wall and the base of the breast.

A DCMREF surface was initially created for all of the patients in this study. The patients were set up while free breathing using the DCMREF surface at a pretreatment imaging session. Setup tolerance was nominally 3 mm/3°, but the real‐time recommended shifts were brought as close to zero as possible at setup. If the port images acquired with the DCMREF surface agreed with the reference images to ≤3mm, the patient was set up on a daily basis with the DCMREF surface. If the port images were not adequate, the patient was repositioned based on the port images, than a VRTREF surface was acquired and used for setup for subsequent fractions. Reasons for acquisition of a VRTREF surface were differences in patient conformation or setup between simulation and treatment. Twenty of the patients were set up with DCMREF surfaces, while 10 were set up with VRTREF surfaces. A region of interest (ROI) is defined on the reference surface used by AlignRT in the registration process. The ROI's used in this work were defined in a similar manner to those used by Shah et al.^(20)^ to include the ipsilateral chest minus the pendulous breast tissue (see Fig. 1). Surface imaging alignment with this ROI was shown by Shah and colleagues to produce setups that agreed with port imaging‐based alignment to <3mm for roughly 85% of the 50 sampled cases.

In this work the ROI's were drawn to include the ipsilateral chest wall and the base of the breast, while excluding any pendulous breast tissue. A review of this ROI at our site showed similar results to those described above. Surface imaging‐based alignment with this ROI agreed with port imaging alignment to <3mm for about 90% of the port images acquired over the treatment of 20 patients. Pendulous breast tissue that might move between fractions will generally show minimal movement with respect to the chest wall and base of breast included in this ROI, and as such this ROI should serve as a good indicator of intrafraction motion.

**Figure 2:**
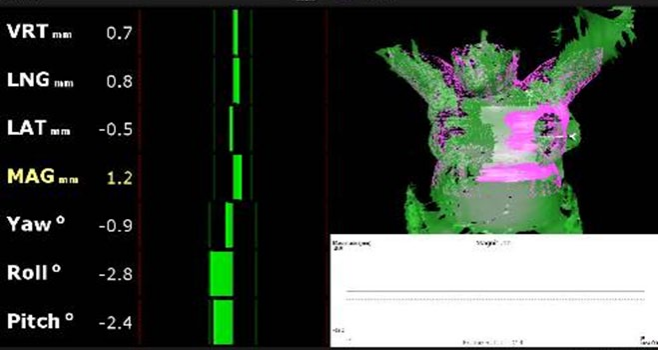
An example of the AlignRT monitoring screen seen during treatment. The real‐time RMS offset along with linear translations and rotations are shown on the left. The bars are green if the suggested shifts are within a predefined tolerance (3 mm and 3° in this case). The reference surface and ROI are shown in pink on the right. The real time AlignRT surface is overlaid on the reference surface in green. At setup, the therapists attempt to minimize the shifts (by minimizing the length of the green bars). Note, the term RMS is used in the text to refer to the MAG shown in the figure.

The patients were set up and monitored throughout treatment using AlignRT in real‐time mode. In real‐time mode, AlignRT displays suggested linear translations (vertical, lateral, longitudinal), rotations (yaw, pitch, roll), and a root mean square of the linear translations (RMS) that will minimize the disagreement of the registration between the reference surface and the real‐time–generated AlignRT surface (Fig. 2), or the displacement of the patient from the machine isocenter. The patients were initially set up by minimizing all of the AlignRT suggested real‐time shifts. Three millimeter and 3° tolerances were used for monitoring. The treating therapists were instructed to adjust the patient position based on AlignRT‐suggested shifts during treatment at their discretion.

The real‐time shifts were recorded to a text file every 0.2–0.4 s depending on the size of the ROI for each monitoring session, where the term monitoring session will be used to refer to a continuous, uninterrupted series of measurements. Most fractions consisted of a single monitoring session.

### C. Data analysis

**Figure 3:**
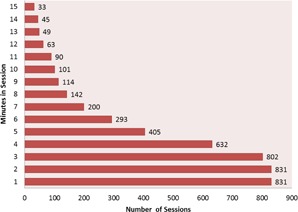
Number of sessions and sampling time.

For data analysis, all monitoring sessions that lasted <2min were discarded. Data points were excluded from the initial setup period and at the end of treatment when the patient was being taken out of the treatment position based on the following criteria: data at the beginning of the text file that had shifts >3mm and data acquired during the final minute of monitoring. For monitoring sessions that lasted longer than 15 min, only the initial 15 min following setup were included in the analysis. This left a total of 831 separate monitoring sessions. The number of monitoring sessions per patient ranged from 17 to 37. The duration of the analyzed monitoring sessions was 4,747 min, with a range of 81 min to 255 min per patient, giving a mean monitoring time per session of about 5.7 min. Figure 3 shows a breakdown of the number of sessions and their durations.

**Figure 4:**
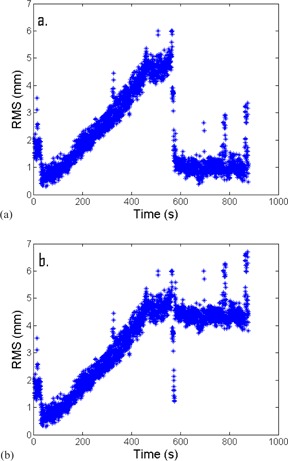
Example of raw RMS measurements (a) as a function of time for a monitoring session that shows therapist correction of patient position around the 600 s time. Example of “uncorrected” RMS data (b), where the data after the intervention shown in (a) have been rescaled to account for intrafraction repositioning (i.e., to give an estimate of patient position if no intrafraction repositioning had occurred).

The number of intrafraction corrections applied by the therapists was retrospectively determined by identifying instances in the monitoring sessions where the RMS values were >3mm for 30 s then abruptly changed to ≤2mm for at least 10 s. These adjustments were then “uncorrected” from the linear shift data used for analysis by scaling the data at time points after the correction by the magnitude of the intrafraction adjustment (see Fig. 4).

Each day the patients were initially positioned to minimize the AlignRT‐suggested real‐time shifts. Rarely did this result in all of the suggested shifts being completely zeroed at the beginning of treatment. The initial positions of the patients at each session were determined by taking the average of the suggested shifts over first the 15 s of monitoring (after all processing described above had been performed). The initial values were subtracted from the monitoring data to yield data that more accurately reflected movement from the initial position during treatment (i.e., data that were less biased by the initial setup position).

The data were analyzed as functions of minutes in the monitoring session and monitoring session number. For each patient, the averages and standard deviations of the linear translations, rotations, and RMSs were calculated over each minute, each session, and over all the sessions. The percent of time for each session that the RMS values were outside of 2 mm, 3 mm, 4 mm, and 5 mm and the maximum consecutive time in each session that the RMS values were outside of 2 mm, 3 mm, 4 mm, and 5 mm were evaluated for each patient.

Statistical analysis of the data was performed to evaluate differences between the means of measurement groups using Students *t*‐tests, with p<0.05 indicating significance. The mean real‐time shifts over all sessions for each individual patient were compared to the mean shifts for all patients.

Correlations between real‐time mean shifts per minute over all sessions (e.g., the shifts from minute 1 of all sessions were averaged) and minutes in the session were evaluated using Pearson's *r* value. Correlation values of r<0.4 were considered weak, 0.4≤r≤0.7 were considered intermediate, and r>0.7 were considered strong. Statistical significance was defined as p<0.05. Correlations between standard deviation of the shifts per minute and minutes in the monitoring session, mean shifts per session and session number, and standard deviation of the shifts per session and session number were also evaluated with Pearson's *r* value.

This work was performed with the approval of the Cone Health Internal Review Board under protocol 1421.

## III. RESULTS

### A. Surface imaging system validation

The mean RMS position of the phantom given by the DCMREF surface was 0.39 mm over the 15 min monitoring period. The range of the measurements was 0.11 to 0.80 mm. Two SDs of the data (95% confidence interval) were 0.22 mm. The mean RMS position of the phantom for the VRTREF surface was 0.20 mm over the 15 min monitoring period. The range of the measurements was 0 to 0.47 mm. Two SDs of the data were 0.12 mm.

The mean RMS disagreement between the expected surface imaging values after couch shifts and the measured values was 0.13 mm for both the DCMREF and the VRTREF surfaces. The range of disagreements for the DCMREF surface was 0–0.42 mm. The range of disagreements for the VRTREF surface was 0–0.37 mm.

The surface imaging system is adequately stable and accurate to monitor millimeter scale positional shifts.

### B. Patient measurements

A total of 127 intrafraction corrections were identified from the 831 monitored sessions. The most interventions during a single monitoring session were 2, which occurred in 14 sessions. Twelve of the patients had corrections made on <10% of their monitoring sessions, while three patients had corrections made on >30% of the monitoring sessions. The “uncorrection” of the data led to changes in the overall mean RMS times of <5% for 13 of the cases and <10% for 23 of the patient cases. The largest change in mean RMS position for an individual patient was 20% (about 0.6 mm). The mean RMS value over all patients changed from 2.23 mm to 2.39 mm when the corrections were accounted for. The majority of the monitoring sessions did not include any intrafraction corrections. For sessions where corrections occurred, the corrections generally were not made at the beginning of a session; therefore, the total amount of data affected was less than 15% (127 of 831 sessions). The resulting analysis of the uncorrected data should provide a reliable representation of patient motion if no intrafraction corrections had been made.

**Figure 5:**
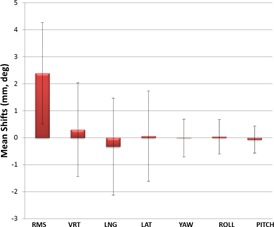
Mean shift values over all patients and sessions.

The mean shift values over all patients and sessions are shown in Fig. 5. The mean RMS value averaged over all patients was 2.39mm±1.88mm. The range of mean RMS values for the patient cases was 1.16 mm to 4.52 mm. The range of RMS standard deviation for the individual patient cases was 0.84 mm to 3.21 mm. The mean vertical displacement over all patients was the largest linear translation at about 0.3 mm. The mean rotations were all <0.1°. The standard deviations for the individual linear translations over all patients range from 1.67 mm to 1.80 mm, while the standard deviations of the rotations range from 0.50° to 0.70°. The full patient motion data are shown in Table 1. The mean position of the patients likely represents movement or drift of the patients away from their initial positions over the span of each monitoring session, while the standard deviations likely reflect a combination of drift, respiratory motion, and other short‐term movements away from the initial position. The relatively small mean values for the linear translations and rotations indicate that there were no systematic offsets in the patient motion, and that motion was likely random about the initial position.

**Table 1:** The mean shifts over all sessions for each of the patients. The bottom row shows the mean shifts for all patients combined.

	*RMS Mag (mm)*		*Vertical (mm)*		*Longitudinal (mm)*		*Lateral (mm)*		*Yaw (deg)*		*Roll (deg)*		*Pitch (deg)*	
*Case*	*mean*	*std*	*mean*	*std*	*mean*	*std*	*mean*	*std*	*mean*	*std*	*mean*	*std*	*mean*	*std*
1	1.78	1.33	0.14	0.84	−0.51	1.57	−0.52	1.11	0.18	0.53	−0.06	0.46	0.00	0.32
2	2.98	2.12	−0.09	2.82	−0.57	1.69	−0.43	1.44	−0.04	0.52	0.05	0.75	0.04	0.58
3	1.16	0.93	0.04	0.67	−0.03	0.85	−0.27	0.99	−0.05	0.48	0.08	0.22	−0.01	0.29
4	2.46	1.54	0.40	2.18	−0.24	1.56	−0.04	1.01	0.07	0.71	0.19	0.44	−0.15	0.23
5	2.58	1.59	0.41	1.52	−0.05	1.77	0.64	1.78	−0.14	0.64	−0.10	0.46	−0.16	0.44
6	2.20	1.81	0.38	1.97	0.16	1.40	−0.60	1.32	−0.12	0.69	−0.39	0.61	−0.06	0.31
7	2.37	1.58	0.57	1.31	−0.01	1.26	1.14	1.78	−0.20	0.95	−0.25	0.91	0.07	0.71
8	2.01	1.43	0.98	1.35	−0.07	0.90	0.65	1.43	0.01	0.49	0.35	0.45	−0.05	0.43
9	4.52	2.21	−0.80	2.35	−1.55	2.96	0.19	2.82	0.12	1.32	0.43	1.43	−0.30	0.79
10	3.21	2.20	−0.01	1.61	−2.23	2.34	−0.13	1.45	0.60	0.61	0.03	0.66	−0.05	0.39
11	2.08	1.95	0.64	1.55	−0.34	1.55	−0.21	1.65	0.00	0.74	0.05	0.59	0.03	0.57
12	1.65	0.84	1.00	0.76	−0.41	0.60	−0.38	1.09	−0.09	0.39	−0.12	0.28	−0.14	0.23
13	1.24	0.89	0.05	0.60	−0.27	0.75	0.19	1.14	−0.17	0.29	0.12	0.36	−0.02	0.22
14	2.71	1.62	−0.36	1.30	0.48	2.08	−0.83	1.69	−0.39	0.52	−0.14	0.65	0.10	0.53
15	2.18	1.39	−0.03	1.22	−0.37	1.60	−0.58	1.47	−0.32	0.97	0.12	0.73	−0.14	0.55
16	3.26	2.04	1.02	1.93	−1.19	1.86	1.33	1.85	0.02	0.91	0.11	0.81	0.31	0.64
17	2.18	1.52	0.45	1.06	−0.21	1.64	0.86	1.50	0.06	0.46	0.09	0.47	−0.27	0.46
18	2.73	1.73	0.82	1.68	1.08	2.09	0.39	1.13	0.08	0.37	0.00	0.34	−0.37	0.50
19	2.91	2.33	0.32	1.66	−0.76	1.70	0.80	2.63	−0.16	0.49	−0.32	0.46	−0.35	0.41
20	2.25	1.72	0.61	1.46	−0.56	1.31	1.02	1.55	0.14	0.37	0.26	0.65	−0.30	0.46
21	1.50	1.10	0.34	1.06	−0.06	1.10	0.14	1.00	0.13	0.35	0.11	0.39	−0.18	0.39
22	2.93	2.03	1.31	1.69	−0.46	1.79	−0.43	2.13	−0.03	0.83	0.34	0.50	0.06	0.37
23	2.19	1.60	0.10	0.98	0.00	1.77	−0.19	1.79	0.11	0.70	−0.09	0.64	−0.21	0.61
24	2.05	1.67	0.36	1.07	−0.16	2.12	0.05	1.10	−0.14	0.63	−0.06	0.55	−0.02	0.39
25	1.86	1.15	0.68	1.28	0.51	1.12	0.29	1.05	0.23	0.49	0.15	0.32	−0.34	0.36
26	2.20	1.21	−0.02	1.47	−0.37	1.69	−0.02	1.06	0.39	0.99	−0.05	0.42	−0.23	0.45
27	2.90	2.04	−0.16	2.27	−0.09	1.67	−0.58	2.06	−0.29	0.46	−0.10	0.35	0.26	0.48
28	1.88	1.02	1.06	1.16	0.33	1.13	−0.23	0.80	0.05	0.39	0.02	0.28	0.04	0.24
29	1.99	1.22	0.88	1.39	−0.31	1.18	−0.15	1.11	−0.12	0.31	0.01	0.35	0.15	0.31
30	3.48	3.21	−0.93	3.17	−0.85	2.71	−0.16	1.85	−0.20	0.82	0.32	0.69	−0.05	0.62
Total	2.39	1.88	0.30	1.74	−0.33	1.80	0.06	1.67	−0.01	0.70	0.04	0.64	−0.07	0.50

Twenty‐nine of the 30 patients showed significantly different mean RMS values from the mean of the total group (p=0). Only one patient had a mean RMS value similar to the entire group (p=0.013). Although the majority of the patients' mean RMS values were statistically different from the group as a whole, only one patient measurement fell outside of 1 SD of the group mean. This indicates that the group mean is a good indicator of the individual patient measurements, with few outlying cases. The statistical differences between the individual means and the group means were a result of the large sample numbers and the relatively tight variances of the individual groups.

**Figure 6:**
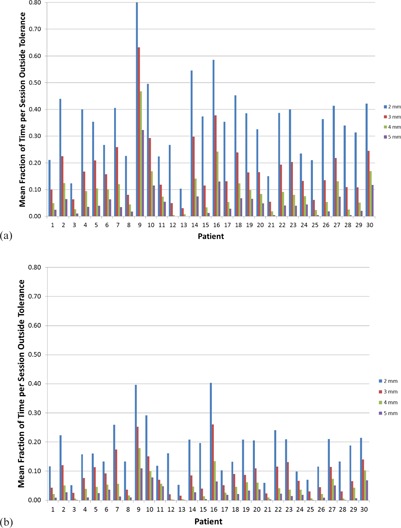
(a) The total time spent outside a defined tolerance per session and (b) the consecutive time spent out of a defined tolerance per session normalized by the session time. Tolerances of 2 mm, 3 mm, 4 mm, and 5 mm are shown for each patient.

The total time a patient spent with their RMS value out of a defined tolerance per monitoring session and the maximum consecutive time a patient spent with their RMS out of tolerance per monitoring session are reported as fractions of the monitoring session time to facilitate interpatient comparison (shown in Fig. 6). Eight patients spent >95% of one monitoring session outside of the 2 mm RMS tolerance. Only two patients spent >95% of a session outside of 3 mm RMS tolerance (one of those patients spent >95% of a session outside of 4 mm RMS tolerance). On average, the patients spent 34%, 17%, 9%, and 5% of the monitoring time outside of 2 mm, 3 mm, 4 mm, and 5 mm RMS tolerances, respectively. They spent average maximum consecutive times of 17%, 9%, 5%, and 3% outside of 2 mm, 3 mm, 4 mm, and 5 mm RMS tolerances, respectively. In only one case was the mean consecutive time outside 5 mm RMS tolerance >10%, and only two cases had mean consecutive times outside 4 mm RMS tolerance >10%. This indicates that the patients are staying within 5 mm, and in many cases 4 mm, on a per case basis with few outliers.

**Figure 7:**
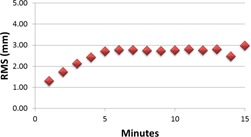
Mean RMS shifts per minute over all patients and sessions. A strong correlation can be seen between minutes in the session and RMS displacement.

The real‐time shifts for all patients averaged over each minute in the monitoring session were evaluated. The mean RMS values show a strong, significant, positive correlation with monitoring time (r=0.73,p=0.002), as shown in Fig. 7. This trend was also evident on a per patient basis, as 22 of the cases had RMS values that showed strong significant positive correlation with time in the monitoring session (r>0.70,p≤0.010) and two cases showed moderate significant positive correlation (r>0.58,p≤0.020). On a per patient basis, the individual linear translations and rotations also showed a trend towards increased movement with time in the monitoring session, with over half of the individual patient cases having strong significant correlation between time and displacement for the linear shifts and rotations (r>0.70,p<0.010). The full data are available in Table 2.

**Table 2:** The mean shifts per minute over all patients and sessions. The bottom two rows show the Pearson's *r* values and p‐values evaluating correlation of each column with time.

	*RMS Mag (mm)*		*Vertical (mm)*		*Longitudinal (mm)*		*Lateral (mm)*		*Yaw (deg)*		*Roll (deg)*		*Pitch (deg)*	
*min*	*mean*	*std*	*mean*	*std*	*mean*	*std*	*mean*	*std*	*mean*	*std*	*mean*	*std*	*mean*	*std*
1	1.30	1.07	0.05	0.93	−0.03	1.13	0.01	0.84	0.00	0.42	0.00	0.34	−0.02	0.31
2	1.72	1.29	0.25	1.16	−0.09	1.34	0.03	1.20	−0.01	0.58	0.00	0.48	−0.09	0.38
3	2.13	1.45	0.40	1.40	−0.14	1.57	0.05	1.42	−0.03	0.67	−0.02	0.57	−0.10	0.47
4	2.43	1.65	0.39	1.62	−0.30	1.72	0.07	1.66	−0.04	0.78	−0.01	0.64	−0.06	0.55
5	2.71	1.83	0.28	1.88	−0.39	1.85	0.00	1.87	−0.06	0.83	0.00	0.75	−0.05	0.59
6	2.77	2.06	0.26	2.03	−0.52	2.08	0.06	1.77	−0.03	0.78	0.11	0.79	−0.04	0.55
7	2.78	2.19	0.20	2.12	−0.60	2.10	0.17	1.77	0.00	0.75	0.13	0.81	0.00	0.58
8	2.73	2.18	0.22	2.08	−0.49	1.99	0.16	1.90	−0.02	0.72	0.12	0.76	−0.03	0.57
9	2.72	2.18	0.37	1.98	−0.46	1.97	0.28	1.98	−0.02	0.71	0.09	0.70	−0.02	0.55
10	2.76	2.27	0.36	2.15	−0.44	1.98	0.11	1.96	0.03	0.75	0.08	0.73	−0.06	0.56
11	2.80	2.22	0.40	2.09	−0.53	2.03	0.15	1.96	0.06	0.74	0.12	0.73	−0.04	0.56
12	2.76	2.31	0.15	2.09	−0.58	1.99	0.27	2.05	−0.01	0.62	0.09	0.70	−0.01	0.53
13	2.80	2.51	0.16	2.04	−0.59	2.18	0.30	2.19	−0.02	0.64	0.09	0.75	−0.01	0.55
14	2.47	2.16	0.18	1.91	−0.24	1.92	0.19	1.81	−0.01	0.65	0.07	0.72	0.00	0.60
15	2.98	2.30	0.16	2.19	−0.44	2.29	0.26	1.96	−0.03	0.69	0.09	0.83	0.00	0.63
r	0.73	0.88	−0.16	0.78	−0.62	0.81	0.84	0.80	0.24	0.19	0.66	0.69	0.68	0.70
p	0.002	0.000	0.569	0.001	0.013	0.000	0.000	0.000	0.398	0.502	0.007	0.004	0.005	0.003

**Figure 8:**
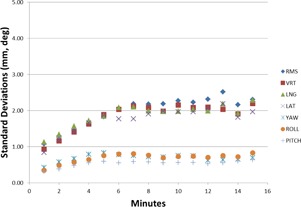
Changes in the standard deviations over the group of all patients as a function of time.

As shown in Fig. 8, the standard deviations of the linear translations for the entire patient group showed strong significant correlations with time (r>0.78,p<0.001). The roll and pitch showed a moderate significant correlation (r>0.68,p<0.005), while the yaw showed no correlation (r=0.19,p=0.502). More than half of the standard deviations for the individual patient cases showed a strong or moderate significant correlation with time.

These results indicate that the patients tend to drift further away from their initial position and they tend to have more short‐term random motion as time in the treatment position increases. Time on the treatment table may be a valid concern for modulated treatment deliveries and should be considered. Drift, or change in absolute position, may be accounted for with careful monitoring and correction, but it will be difficult to counteract increased random motion with increasing time on the treatment table.

Patient motion was also evaluated as a function of monitoring session number (the full dataset is shown in Table 3). The mean lateral and roll showed weak significant correlations with session number (r=−0.38,p=0.020). No other significant correlations between translations and rotations were observed. Similarly, on a per patient basis the RMS and individual linear translations showed little meaningful correlation with session number, where the correlation between RMS and session number only reached significance (p<0.050) for 3 of the 30 patients. It appears that session number does not play an important role in patient motion.

**Table 3:** The mean shifts per session. The bottom two rows show the Pearson's *r* values and p‐values evaluating correlation of each column with monitoring session number.

	*RMS Mag (mm)*		*Vertical (mm)*		*Longitudinal (mm)*		*Lateral (mm)*		*Yaw (deg)*		*Roll (deg)*		*Pitch (deg)*	
*Session*	*mean*	*std*	*mean*	*std*	*mean*	*std*	*mean*	*std*	*mean*	*std*	*mean*	*std*	*mean*	*std*
1	3.27	2.00	0.73	2.05	−0.67	2.24	0.29	2.09	−0.02	0.80	0.13	0.78	0.15	0.56
2	2.80	1.72	0.49	1.68	−0.27	1.83	0.34	2.05	−0.03	0.73	0.15	0.76	−0.12	0.54
3	2.13	1.68	0.37	1.82	−0.41	1.53	0.04	1.18	−0.03	0.71	0.03	0.54	−0.03	0.53
4	2.18	1.57	0.31	1.45	−0.08	1.71	0.11	1.44	−0.03	0.67	−0.01	0.52	−0.06	0.51
5	2.26	1.93	0.20	1.68	−0.65	1.96	0.23	1.29	0.04	0.78	0.02	0.77	−0.02	0.49
6	2.57	1.96	−0.45	2.28	−0.07	1.61	0.46	1.50	−0.09	0.95	0.16	0.84	−0.11	0.64
7	2.01	1.38	0.49	1.17	−0.07	1.58	−0.06	1.35	−0.06	0.53	0.05	0.52	−0.13	0.44
8	2.11	1.64	0.39	1.28	−0.27	1.85	−0.10	1.35	−0.03	0.72	0.03	0.64	−0.01	0.54
9	2.18	1.87	0.32	1.48	−0.12	2.00	0.04	1.40	0.02	0.90	−0.06	0.62	−0.02	0.53
10	2.23	1.72	0.34	1.45	−0.22	1.88	0.02	1.46	0.21	0.85	0.14	0.50	−0.10	0.48
11	2.43	1.97	0.27	2.02	−0.31	1.55	−0.15	1.77	−0.12	0.69	−0.04	0.81	−0.13	0.48
12	2.44	1.62	0.50	1.65	−0.26	1.80	0.07	1.51	0.21	0.78	0.09	0.65	−0.08	0.52
13	2.09	1.63	0.28	1.21	−0.15	1.76	−0.12	1.54	−0.06	0.64	0.11	0.64	−0.11	0.40
14	2.09	1.64	0.13	1.51	−0.43	1.57	0.06	1.46	−0.01	0.84	−0.17	0.82	−0.11	0.42
15	1.81	1.25	0.42	1.16	−0.25	1.24	0.05	1.31	0.02	0.54	0.03	0.51	0.02	0.40
16	2.95	3.09	−0.58	2.82	−0.98	2.50	−0.31	1.63	0.14	0.68	0.09	0.54	−0.23	0.54
17	2.93	2.22	0.27	2.10	−0.65	2.04	0.48	2.05	−0.08	0.58	−0.10	0.56	−0.16	0.62
18	2.10	1.49	−0.06	1.34	−0.11	1.32	0.03	1.76	−0.02	0.49	0.00	0.46	−0.08	0.42
19	2.02	1.48	0.29	1.11	−0.29	1.48	−0.13	1.63	0.04	0.68	0.11	0.69	−0.12	0.41
20	1.94	1.44	0.50	1.07	−0.08	1.59	0.06	1.38	−0.14	0.65	−0.08	0.50	−0.16	0.42
21	2.41	1.99	0.45	1.28	−0.50	1.97	0.56	1.87	0.03	0.58	0.29	0.79	−0.10	0.46
22	2.56	2.20	0.79	1.41	−0.39	2.09	0.36	2.03	0.15	0.73	0.12	0.59	−0.04	0.53
23	1.89	1.36	0.19	1.02	−0.48	1.56	−0.07	1.30	0.02	0.52	−0.01	0.34	−0.08	0.35
24	2.47	2.09	0.50	1.66	−0.76	1.60	−0.40	2.04	−0.04	0.66	0.15	0.56	−0.11	0.44
25	2.50	1.75	0.36	1.86	0.43	1.57	0.25	1.74	−0.03	0.74	0.07	0.55	−0.24	0.65
26	2.12	1.55	0.60	1.25	−0.40	1.16	0.24	1.86	0.09	0.55	−0.02	0.51	−0.10	0.44
27	1.77	1.35	0.28	1.28	−0.32	1.10	0.05	1.40	−0.07	0.45	0.03	0.49	0.02	0.29
28	2.21	1.55	1.07	1.63	0.34	1.29	−0.71	1.11	−0.01	0.38	−0.24	0.56	−0.10	0.30
29	2.06	1.48	0.07	1.47	−0.19	1.31	0.07	1.58	−0.05	0.39	−0.02	0.48	−0.03	0.32
30	2.40	2.02	0.02	1.05	−1.06	2.26	−0.85	1.34	−0.21	0.47	0.00	0.37	0.14	0.45
31	2.57	1.65	0.13	1.43	−0.42	1.71	−0.81	1.88	−0.27	0.57	−0.02	0.48	−0.12	0.52
32	3.35	2.66	−1.57	3.31	−0.51	1.44	−0.59	1.49	−0.18	0.43	−0.21	0.72	0.08	0.45
33	1.68	1.12	0.23	0.95	0.17	1.09	0.08	1.38	−0.08	0.30	0.07	0.33	−0.12	0.27
34	1.67	1.29	0.52	1.02	0.31	1.32	−0.20	1.13	0.00	0.56	−0.08	0.34	−0.08	0.40
35	1.64	1.27	0.40	1.08	0.38	1.17	0.19	1.19	−0.10	0.39	−0.08	0.40	−0.06	0.35
36	1.92	1.20	−0.44	0.95	−0.08	0.86	−0.42	1.76	0.07	0.32	−0.19	0.61	−0.22	0.32
37	1.78	1.35	0.15	1.11	0.42	1.22	0.26	1.42	0.06	0.28	0.03	0.51	−0.13	0.32
r	−0.32	−0.23	−0.20	−0.27	0.29	−0.53−0.38	−0.11	−0.20	−0.77	−0.38−0.54−0.13	−0.61
p	0.055	0.168	0.245	0.113	0.079	0.001	0.020	0.534	0.247	0.000	0.020	0.001	0.426	0.000

## IV. DISCUSSION

This work represents the most thorough investigation of intrafraction breast motion reported to date, looking at over 800 sessions and more than 4,700 min of monitoring. The 3D surface imaging data support findings by other authors that have evaluated respiratory motion and position drift with intermittent or shorter sampling courses. A review of 13 studies that evaluated breast intrafraction motion with port imaging showed a range of intrafraction motion of 0.7–3.2 mm,^(24)^ while a study using continuous tracking of an external marker showed ~3mm of intrafraction motion.^(25)^ This is in good agreement with the mean RMS displacement from isocenter of 2.39mm±1.88mm found in this work. Several groups have shown respiratory motion on the order of 1 mm using combinations of 4D CT, fluoroscopy, and fiducial markers,^25^, ^26^, ^27^, ^28^ which is also in reasonable agreement with our RMS standard deviation of 1.88 mm that accounts for positional drift over a prolonged monitoring period, in addition to respiratory motion.

Park et al.^(26)^ suggested a PTV margin of 6 mm for conformal breast radiotherapy based on their evaluation of motion using fiducial markers (compared to a 10 mm margin when aligning to bony anatomy) that accounted for average interfraction and intrafraction motion plus 2 SDs. Evaluation of our RMS displacements as a function of time show that a 6 mm margin would be sufficient to account for 2 SDs of motion for treatments under 4 min in length. The work by Park et al.^(26)^ used a pre‐ and post‐treatment 4D CT to evaluate motion. This likely represents a shorter sampling period that does not encounter the positional drift seen over longer monitoring sessions. Our measurements show that, as treatment times increase, the PTV margin would need to be increased to account for 2 SDs of motion (e.g., a margin of 6.4 mm would be needed at 5 min, 7.3 mm at 10 min, and 7.7 mm at 15 min). For longer treatment times, attempts should be made to correct for positional drift or PTV margins should be adjusted accordingly.

All of these measurements pertain specifically to our institution and to our implementation of 3D surface imaging. Our overall RMS value averaged over all patients was 2.39 mm, which is (not unexpectedly) just lower than the 3 mm tolerance we set for treatment delivery. The results are also affected by the amount of repositioning that the therapists perform during treatment. Repositioning occurred during roughly 14% of the monitoring sessions. It was noted earlier that several sessions were conducted with the patient outside of 3 mm tolerance for the majority of the treatment session. Therapists were encouraged to reposition the patient when AlignRT showed RMS values above the 3 mm tolerance, but this was not strictly monitored or enforced, as larger deviations have historically been deemed acceptable in our institution. We feel it is likely that setting a stricter tolerance and/or conducting more regular monitoring of deviations from our defined tolerance levels would result in even smaller and more consistent RMS values that would help establish a high level of confidence in the accuracy of modulated IMRT or electronic compensated breast treatments. Further studies with tolerance criteria specific to the alignment and positioning needs of a treatment technique, along with mandatory adherence to the criteria, are the next phase of this project.

These measurements reflect the position of the patient surfaces throughout treatment, but they do not provide information about internal anatomy. More research should be done to determine if a correlation between the breast surface and lung or heart position exists.

## V. CONCLUSIONS

With the above considerations in mind, it is reasonable to conclude that it is possible to deliver modulated or reduced field treatments within 8 mm of the planned position for a free‐breathing breast patient without surface imaging for treatments lasting less than 15 min. Smaller margins may be appropriate for shorter treatments or patients who are actively monitored with surface imaging. Treatment time should be taken into account when creating margins for patient positioning uncertainty (i.e., PTV margins). Additionally, efforts to reduce time on the treatment table, while maintaining the quality of treatment, can reduce patient motion and provide a more spatially accurate treatment delivery. Investigation into ways to prevent movement or drift over a long treatment may also be warranted.

## ACKNOWLEDGMENTS

The authors would like to thank Peggy Wynn at the Cone Health library and the group at VisionRT for helping to set up acquisition of the real‐time shift information.
